# Role of Notch Receptors in Hematologic Malignancies

**DOI:** 10.3390/cells10010016

**Published:** 2020-12-24

**Authors:** Laura Gragnani, Serena Lorini, Silvia Marri, Anna Linda Zignego

**Affiliations:** MASVE Interdepartmental Hepatology Center, Department of Experimental and Clinical Medicine, University of Florence, Center for Research and Innovation CRIA-MASVE, 50134 Firenze, Italy; laura.gragnani@unifi.it (L.G.); serena.lorini@unifi.it (S.L.); silvia.marri@unifi.it (S.M.)

**Keywords:** Notch, Notch receptor, Notch signaling, T-cells, B-cells, leukemia, lymphoma, hematological malignancies

## Abstract

Notch receptors are single-pass transmembrane proteins that play a critical role in cell fate decisions and have been implicated in the regulation of many developmental processes. The human Notch family comprises of four receptors (Notch 1 to 4) and five ligands. Their signaling can regulate extremely basic cellular processes such as differentiation, proliferation and death. Notch is also involved in hematopoiesis and angiogenesis, and increasing evidence suggests that these genes are involved and frequently deregulated in several human malignancies, contributing to cell autonomous activities that may be either oncogenic or tumor suppressive. It was recently proposed that Notch signaling could play an active role in promoting and sustaining a broad spectrum of lymphoid malignancies as well as mutations in Notch family members that are present in several disorders of T- and B-cells, which could be responsible for altering the related signaling. Therefore, different Notch pathway molecules could be considered as potential therapeutic targets for hematological cancers. In this review, we will summarize and discuss compelling evidence pointing to Notch receptors as pleiotropic regulators of hematologic malignancies biology, first describing the physiological role of their signaling in T- and B-cell development and homeostasis, in order to fully understand the pathological alterations reported.

## 1. Introduction

The Notch gene was first identified in Drosophila [1] as a key developmental gene [2]. Notch receptors are single-pass transmembrane proteins which play a critical role in cell-fate decisions and have been implicated in the regulation of many developmental processes [3]. The Human Notch family comprises of four receptors (Notch 1 to 4) and five ligands— of which are members of the Delta-like (DLL1, DLL3 and DLL4) and the Jagged (JAG1 and JAG2) family [3]. Notch receptors transduce short-range signals by interacting with the transmembrane Delta-like and Jagged ligands on neighboring cells.

The Notch receptors span the cell membrane with extracellular and intracellular domains. Ligands that bind to the Notch extracellular domain result in the initiation of the sequential receptor proteolytic cleavages. In fact, an ADAM-family metalloprotease called ADAM10, cleaves the receptor just outside the membrane and the Notch extracellular domain (NECD) is released [4]. This induces γ-secretase to cleave the transmembrane region at the S3 site, releasing the Notch intracellular domain (NICD) thereby entering the cell nucleus and triggering gene expression [5]. In the nucleus, NICD forms a ternary complex with the DNA-binding protein CBF1/RBPjk/Su(H)/Lag1 (CSL) that helps recruit the adaptor protein Mastermind-like to activate target gene expression [6,7]. During the transcriptional activation process, NICD is phosphorylated on its PEST domain and targeted for proteasome-mediated degradation by ubiquitin ligases known as FBXW7. This limits the half-life of a canonical Notch signal [8].

Although Notch signaling can regulate rather basic cellular processes such as differentiation, proliferation and death, it is also involved in hematopoiesis and angiogenesis [9,10]. Increasing evidence suggests that Notch pathways are involved and frequently deregulated in several human malignancies [11], contributing to cell autonomous activities that may be either oncogenic or tumor suppressive [11].

Notch signaling plays an active role in promoting and sustaining a broad spectrum of lymphoid malignancies [12,13,14]. In addition, mutations in the Notch family members are present in several disorders of T and B-cells [11,13,15] and are responsible for altering the related signaling [12].

This review will cover the main aspects of Notch’s involvement in T and B-cell malignancies, starting with the physiological mechanisms through which Notch signaling regulates normal T and B lymphocyte development and functions, in order to accurately discern how pathway deregulation and genetic mutations influence the transition to malignancy.

## 2. Notch 1

### 2.1. Physiology of Notch 1 Signaling in the Immune System Cells

Notch 1 is one of four Notch receptors expressed in mammalians. Among the five ligands, DLL4 has a higher affinity than DLL1 and JAG1 [16] and it is responsible for Notch 1 activation in the thymus of murine models [17,18]. DLL4-Notch 1 interaction is crucial in endothelial cell communication in response to vascular endothelial growth factor (VEGF) to balance tip and stalk cells in sprouting events [19].

Notch 1 is expressed in hematopoietic stem cells (HSC) and is required for their maturation, even though knockout experiments did not reveal alterations in HSC maintenance [20]. Mice with induced loss of function of Notch 1 showed blockage in T-cell development from early progenitors, before the expression of lineage surface markers [21]. In addition, Marıa J. Garcia-Leon and colleagues demonstrated that a tight regulation of Notch ligand expression in the diverse thymus region drive T-cell development [22]. In particular, the DLL4 ligand is specifically expressed in the thymus cortex epithelial cells during the embryonic phase and is downregulated in the adult thymus when the full T-cell repertoire is completed, confirming once more its pivotal role in Notch 1 induced T-cell development [22].

T-cell lineage can be distinguished into two subsets: γδ and αβ T-cells, which express different surface receptors [23]. Both T-cell subsets develop from common precursors [24]: γδ T-cells are the first to appear in the thymus during fetal development and their relative proportion decreases while αβ T-cells increase [25]. In vitro experiments with the earliest human intrathymic precursors highlighted the role of Noch1 signaling in the development of γδ T-cells [26]. Conversely, it has been shown that αβ T-cells require low levels of Notch activation [27]. Moreover, the zinc finger transcription factor Bcl11b is required for both T-cell commitment and for αβ T-cells specialization [28,29].

In fact, interaction between Notch 1 and its ligands, DLL4 and JAG2, leads γδ T-cell development through the repression of Bcl11b expression [30]. Dolens and colleagues proposed a Notch dependent temporal expression of Bcl11c. In the early critical phase of T-cell development, Notch induced the expression of the BCL11B gene while in the subsequent differentiation of γδ T-cells, induced the expression of miR-17, microRNA from the miR 17-92 cluster, which inhibits BCL11B [30]. Thus, BCL11B expression is upregulated and drives the differentiation in αβ T-cells and it is downregulated in γδ T-cells.

In addition, Ciofani et al. demonstrated that, in the thymus, the interaction between Notch 1 and Dll1 sustained pre–T-cell survival after β selection by promoting glucose metabolism through the PI3K-AKT axis [31]. Wang Q et al. reported that Notch 1 cofactor Zmiz1 is involved in the early maintenance of T-cell progenitors and in the development of T-cell lineage. Zmiz1 is probably required for normal β-selection and, in general, it is important in Notch 1-Myc-related thymocyte proliferation [32].

While the involvement of Notch 1 in T-cells has been well-investigated, its role in B-cells remains mostly unknown. Using mice models and primary mouse B-cells, Kang J.A. and collaborators showed that Notch 1 overexpression increases marginal zone B-cell numbers indicating its role in proliferation. In differentiated B-cells, Notch 1 signaling increases after the B-cell receptor binds to the antigen [33]. Notch 1 knocked-down B-cells showed a decrease in antibody production however it was reverted in splenocyte cultures where different cell types were present [34]. This suggests that Notch 1, or its ligand in other immune cells, could upregulate pathways in Notch 1-defecting B-cells [34].

Furthermore, Notch 1 target genes are involved in cell cycle, growth and survival such as c-MYC [35] and the transcription factor HES1 [36].

The role of Notch 1 in the differentiation of immune cells makes it a highly studied gene in hematologic malignancies, but it is also implicated in a wide range of solid tumors, concerning growth and progression, such as melanoma [37], intrahepatic cholangiocarcinoma [38], prostate cancer [39] and osteosarcoma [40].

### 2.2. Notch 1 in T-Cell Acute and Chronic Lymphoblastic Leukemia

Since Notch 1 is a key factor in T-cell development, it is clear that it has been well investigated in T-cell diseases such as acute lymphoblastic leukemia (T-ALL). The first hint of its involvement in T-ALL pathogenesis was the discovery of (7;9) translocation, a mutation that disrupts the Notch 1 gene, fusing the 3′ end portion encoding its intracellular domain (ICN) to the enhancer and promoter elements of the T-cell receptor (TCR) [41]. This results in the overexpression of a constitutively active form of Notch 1, activating genes that inhibit cell differentiation [41]. In fact, the abnormal Notch 1 signaling consequent to t(7;9) supports the growth of human translocated cell lines [42]. Less macroscopic mutations, although directly activating, were then found in the extracellular heterodimerization domain (HD) and/or the C-terminal PEST intracellular domain of the Notch 1 gene [43] and in the extracellular juxtamembrane region [44] in about 60% and 30% of T-ALL cases, respectively.

**Figure 1:**
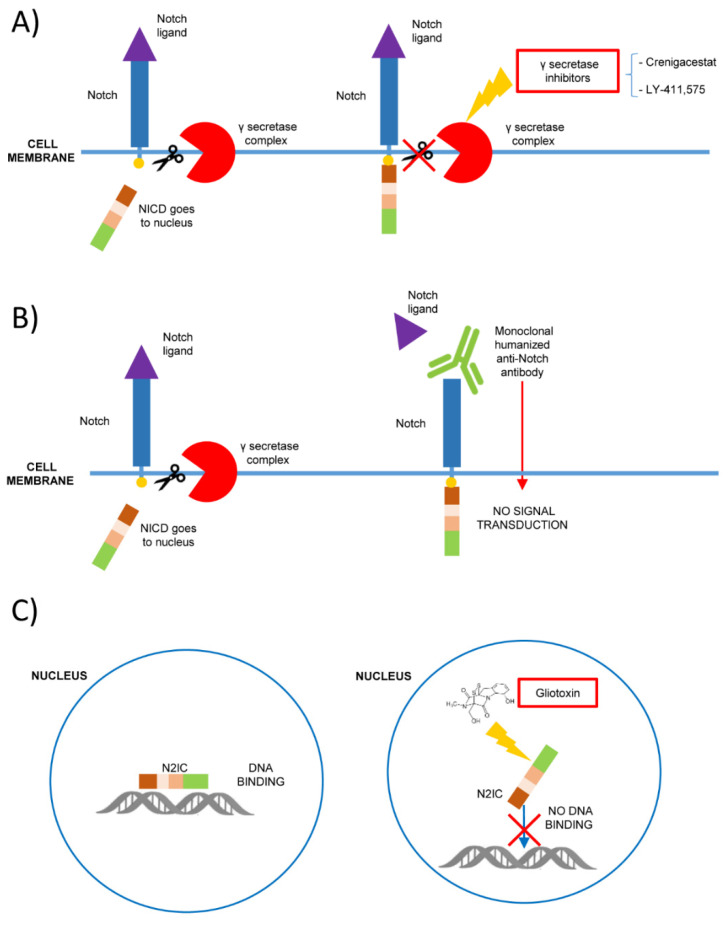
Mechanisms of Notch signaling inhibition as possible therapeutic strategies in hematological malignancies. (**A**) The inhibition of γ secretase-complex (i.e., with Crenigacestat or LY-411,575) prevents the proteolytic cleavage of Notch intracellular domain (NICD), blocking its signaling; (**B**) the binding with monoclonal humanized antibodies prevents Notch ligand interaction and the signaling activation; (**C**) gliotoxin is a specific Notch 2 inhibitor that binds Notch 2 intracellular domain (N2IC) blocking the DNA binding and preventing the target expression.

In this context, Notch 1 blocking molecules such as γ-secretase inhibitors (GSIs) could be of aid in T-ALL treatment [44] (Figure 1). γ-secretase is a protease complex that cleaves the transmembrane protein that has been studied as a target therapy for Alzheimer’s disease [45]. As Notch 1 is involved in different cancers, GSIs have been proposed as a potential drug for different solid tumors and T-ALL [46].

One of the major biases concerning the use of GSIs (i.e., LY-411,575) as therapeutic agents is the non-selective targeting of abnormal Notch pathways that determine an indiscriminate impairment of many physiological processes [47,48]. An in vivo analysis of GSIs targeting presenilin-1, a subclass of γ-secretase overexpressed in T-ALL cells, showed a 60% reduction in splenomegaly and up to a 40% reduction in leukemic cell infiltration, suggesting that this molecule could pose a means of controlling the issue of a widespread, untargeted effect [49]. Despite these promising results, GSIs generated low therapeutic response rates in early clinical trials with severe gastrointestinal adverse events such as intractable diarrhea and skin disorders due to the involvement of Notch 1 in keratinocyte differentiation and metabolism [50].

Recently, the results of a phase I clinical trial for the use of the Notch inhibitor Crenigacestat in T-ALL were published [51]. It was a nonrandomized, open-label, dose-escalation, phase 1 trial in adults with relapsed/refractory T-ALL or T-cell lymphoblastic lymphoma (T-LBL). Crenigacestat prevents the cleavage of NICD and it was used in combination with dexamethasone with the aim of reducing adverse events [51] (Figure 1). The study led to the administration of the correct drug dose in phase II of the trial, although clinical efficacy was affected by gastrointestinal events despite the use of steroids [51]. Moreover, patients harbored different mutations and the sample was heterogeneous. In fact, only 7/36 patients carried Notch 1 mutations, which, as previously stated, are present in almost 60% of T-ALL. In total, two phase I clinical trials on the use of Crenigacestat on solid tumors showed similar limitations [52,53], confirming that further studies are needed to increase the safety and efficacy of this treatment.

The work of Palomero and collaborators showed that a parallel pathway exists for those who did not require Notch 1 activating mutations and this could explain the failure of GSIs [54]. The phosphatase and tensin homolog (PTEN) protein is known to be a critical negative regulator of PI3K-AKT signaling that promotes cell growth, proliferation and survival [55]. The transcriptional factor Hes1 is one of the Notch 1 targets and acts as a negative regulator of PTEN synthesis. Indeed, Hes1 binding to a PTEN promoter decreases protein levels. This is a positive effect of the PI3K-AKT pathway. In normal thymocytes this signaling regulates development, while in aberrant T-cells it promotes tumor progression. PI3K-AKT could be over stimulated either by Notch 1 activating mutations or by a functionally compromised mutated PTEN. Indeed, PTEN alteration was found in 8% of T-ALL samples and the loss of protein function was demonstrated in 17% of T-ALL and lymphoma samples [56].

O’Neil et al. found mutations in the ubiquitin ligase F-box and WD40 repeated domain containing-7 (FBXW7) gene that could be a possible cause of GSIs failure [57]. In fact, the FBXW7 protein is involved in NICD turnover, resulting in an increase of NICD and c-MYC protein levels [58]. The authors analyzed the response of GSIs treatment in 20 T-ALL cell lines and only five were GSIs sensitive while the remaining did not show any alteration in cell cycle, viability and apoptosis rate. A total of seven GSIs resistant-cell lines expressed high levels of NICD without carrying activating mutation in the Notch 1 gene while the FBXW7 coding region had heterozygous/homozygous mutations. Moreover, the authors found genetic FBXW7 alteration in 8.6% of primary T-ALL samples [57] as confirmed in subsequent studies [59,60,61].

Several studies have been dedicated to finding other therapy targets upstream of Notch 1 pathways, such as transcriptional factors [62] or ubiquitin-peptidase [63].

Recent in vitro and in vivo experiments with xenografts derived from patients found that both GSI-sensitive and GSI-resistant T-ALL are susceptible to navitoclax, an inhibitor from the BCL2 family of anti-apoptotic proteins suggesting the possible use of this drug in therapeutic treatment [64].

The crosstalk with the microenvironment mediated by the cell surface receptor CXCR4 influences Notch 1-signaling [65]. Thus, murine models of Notch 1-induced T-ALL showed that CXCR4 is required for the migration of T-ALL cells. The expression of CXCR4 is induced by calcineurin, a serine/threonine protein phosphatase that activates the T-cells. Moreover in vitro and in vivo CXCR4 silencing increased cell death, altered cell-cycle progression and inhibited migration [65]. Pitt and colleagues performed in vivo experiments observing that CXCL12, an endogenous ligand of CXCR4, is secreted by various cell type including endothelial cells which could represent a niche for T-ALL cells [66]. Silencing CXCL12 in vascular endothelial cells reduced tumor burden and migration in mouse models. Furthermore, the authors showed that targeting CXCR4 both in mice and in human xenograft models suppressed leukemia progression [66]. These findings suggest a novel therapeutic target for pediatric/adult T-ALL, which is difficult to treat.

However, bone marrow samples isolated from four patients were analyzed using single-cell sequencing and showed that mutations in the Notch 1 gene were late events in the T-ALL progression [67].

The role of the transcription factor NF-kB pathway is central for physiologic proliferation and survival of lymphocytes and, when altered, it can be involved in hematologic malignancy pathogenesis [68,69]. In vitro experiments demonstrated that NICD directly binds to the promoters of NF-kB factor increasing the protein expression [70]. Thus, in human T-ALL harboring Notch 1 activating mutation, NF-kB was consequently overexpressed.

Furthermore, Notch 1 prolongs NF-kB activity by physically retaining the protein into the nucleus [71].

The antiapoptotic protein Bcl2a1 is upregulated by NF-kB [72] and it has been shown to prolong the survival of pre-T-cells in the thymus under the stimulation of the pre-T-cell Receptor mediated by NF-kB [73]. In this context, the overexpression of Notch 1 boosts the NF-kB pathway with the consequent overexpression of Bcl2a1 contributing to cell transformation.

A recent study highlights the role of Abs2 (ankyrin repeat-containing protein with a suppressor of cytokine signaling box 2) protein in the Notch 1/NF-kB axis [74]. Abs2 is upregulated by Notch 1 and induces IκBα degradation, a nuclear factor that inhibits NF-kB [74]. When Abs2 is upregulated under the control of Notch 1, IκBα levels decrease, preventing NF-kB inhibition.

Notch 1, Notch 2 and their ligands Jag1 and Jag2 are constitutively expressed in B-cell chronic lymphocytic leukemia (CLL) and confer resistance to apoptosis in malignant B-cells [75]. A whole genome sequencing on four CLL human samples revealed the presence of Notch 1 somatic mutations which generated a truncated protein [76]. Further genetic analysis of 255 CLL patients showed that these alterations are present in 10% of cases and generate a stable and activated form of the protein [76]. Moreover, patients carrying these mutations had a more advanced stage of CLL and a higher risk of undergoing transformation into diffuse large B-cell lymphoma (DLBCL) than patients with CLL without genetic changes. Notch 1 mutations in CLL patients seem to be useful as a predictive risk factor of malignancy evolution [77,78,79,80].

The Notch 1 pathway appears to be a critical regulator of CLL evolution and even in the absence of mutation it is possible to find high levels of the protein. Close and collaborators showed that FBXW7 mutations reduce NICD degradation and lead to its activation in CLL cells [81]. The Notch family was also studied in bone marrow (BM) cells as it would seem that the BM microenvironment is responsible for de novo drug resistance in myeloma and leukemia cells [82]. Multiple myeloma (MM) cells are localized in the BM where the Notch 1-JAG1 interaction results in significant apoptosis protection due to the accumulation of p21WAF/Cip, which is a regulator of cell cycle progression [82].

Recent advances regarding DLL4-Notch 1 signaling in CLL revealed its importance as a promising therapeutic target [83]. In fact, DLL4 is widely expressed in lymph-node histiocytes and its interaction with Notch 1 increases cell proliferation, migration and neo-angiogenesis, in particular in CLL cases carrying Notch 1 mutations. This binding could be antagonized using specific anti-Notch 1 monoclonal antibodies [83]. These data also require further investigation, but it seems conceivable that the use of monoclonal antibodies appears to be a possible therapeutic strategy in CLL patients with mutated Notch 1 and aggressive disease [84] (Figure 1).

### 2.3. Notch 1 and B-Cell Malignancies

The involvement of Notch 1 in B-cell disorders was also investigated. Rosati E and co-workers demonstrated a high expression of Notch 1, Notch 2, JAG1 and JAG2, in malignant B-cells isolated from 25 patients affected by B-cell chronic lymphocytic leukemia (B-CLL) but not in normal B-cells [75]. In fact, the mRNA levels were comparable between patients and healthy subjects and the differences in protein expression levels may be attributable to an altered turnover process. Mature B lymphocytes enriched with Notch 1 express high levels of interleukin 33 (IL-33) which is responsible for increased regulatory (Treg) and helper T (Th) cells and their specific cytokines production, suggesting the role of Notch 1 signaling in the B lymphocyte-related T-cell stimulation [85].

Notch 1 mutations were also found in 5–10% of mantle cells lymphoma (MCL) and are associated with a poor prognosis [86,87]. In MCL harboring Notch 1 mutation, an overexpression of the Notch 1 pathway by DLL4 interaction was observed [88]. This sustained stimulation enhances tumor cell migration and angiogenesis and could be blocked in vitro by a humanized monoclonal Notch 1 antibody. Other kinds of mutations were identified in specific MCL variants with a severe prognosis [89,90] (Figure 1). These latter studies contribute to the genetic profiling of MCL and could aid in diagnostics, surveillance, and potentially the more precise usage of therapeutic drugs by efficient screening of biomarkers in a poor prognosis lymphoma type.

Additionally, genetic alterations in Notch 1 signaling, with the subsequent overexpression and activation of the Myc pathway, were present in 30% of cases relating to Richter Syndrome, which is an aggressive lymphoma evolving from CLL [13].

Recently, a variant in the extracellular domain of Notch 1 that provides a growth advantage to cells and confirms the suitability of the Notch pathway as a second-line druggable target was found in a severe form of lymphoma—the anaplastic large cell lymphoma [91].

Due to their differential role in B and T-cell physiology, the Notch 2 member seems to be the most involved in lymphomagenesis even if a few Notch 1 signaling alterations were described in low as well as high grade lymphomas.

Notch 1 mutations were found in 5% of splenic marginal zone lymphomas (MZLs) [92].

Arcaini et al. analyzed 46 Hepatitis C Virus (HCV) positive patients with DLBCL and 64 DLBCL without viral infection [93]. They found Notch 1 mutations in 4% of DLBCL-HCV positive patients while none of the HCV negative ones were affected. Patients with genetic alterations are characterized by a worse survival rate and in this context, Notch 1 could represent a prognostic marker of malignancy progression.

The involvement of Notch 1 in mucosa-associated lymphoid tissue (MALT) lymphomas was also investigated. In fact, Mensah and collaborators examined the mutational status of PEST domain in 23 cases of MALT lymphomas and no alterations were found, [94] although in a further study, mutations in Notch 1 and Notch 2 were found in 8% of analyzed cases [95]. More recently, next generation sequence analysis of five cases of MALT lymphomas revealed the presence of Notch 1 alteration in three patients [96], but further studies are needed to explore the contributions in this lymphoma subtype.

**Figure 2:**
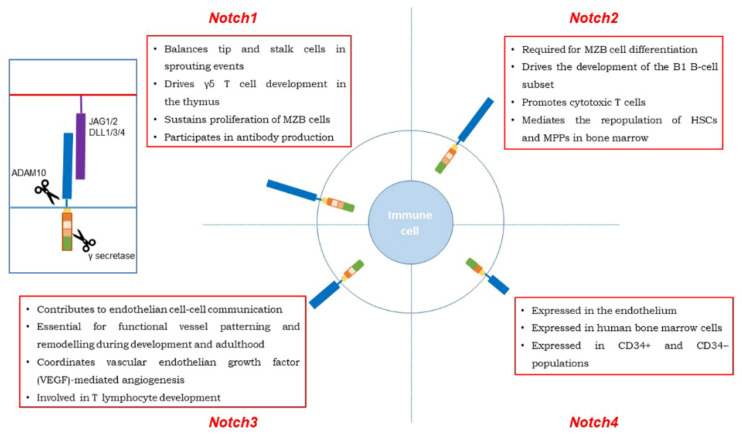
Scheme of all Notch receptors and their physiologic roles.

Moreover, Notch 1 together with Notch 2 mutations were found in a small percentage of follicular lymphomas (FLs) and are characterized by a higher incidence of splenic involvement [97]. The biologic effects in the physiology of the immune system cells are described in Figure 2, while the main Notch 1 mutational events and pathway alterations in leukemias and lymphomas are summarized in Table 1.

## 3. Notch 2

### 3.1. Physiology of Notch 2 Signaling in the Immune System Cells

The Notch 2 molecules are synthesized as single precursor proteins that are cleaved in the Golgi to become functional heterodimeric receptors present on the cell surface [98].

As previously explained, the interaction of Notch receptor with ligands results in further proteolytic cleavage, with the release and nuclear translocation of the NICD which functions as a transcriptional regulator [113].

NICD enters the nucleus and seeks coactivators such as Mastermind-like proteins (MAML 1-3). Together with the recombinant signal-binding protein for immunoglobulin kappa J region (RBPJ) transcription factor, they form the transcription complex accountable for the induction of Notch target genes [114].

Notch 2 is expressed in many cell types in the hemato-lymphoid compartment with specific roles in the development and differentiation of immune system cells.

In the spleen, Notch 2 activation is crucial for marginal zone (MZ) B-cells growth [115] and for the dendritic cells (DCs) differentiation [116].

Mature splenic B-cells are divided into two types of B-cells, follicular B (FOB) cells and MZB-cells [117]. Immature B-cells, developing in the bone marrow, migrate to the spleen and initially differentiate into type 1 (T1) transitional B-cells that subsequently become type 2 (T2) transitional B-cells [118]. Thereafter, these progenitors differentiate into the others two cellular types, MZB and FOB-cells. Notch 2 and no other Notch family member plays a role in the step from T2 B-cells to MZB and its activation is mediated by the DLL1 ligand [115]. DLL1 binding to the Notch 2 receptor triggers the Notch 2 signaling cascade through ADAM10-mediated cleavage and the conditionally targeted deletion of Notch 2 results in a defect of the MZB-cells [115].

Furthermore, it was recently discovered that Notch 2-dependent transcription is active in classical DCs and that it is selectively required for support of the germinal center reaction [119]. In fact, while Notch 2-dependent classical DCs are required in vivo for the induction of follicular Th cells [119], Notch 2 activation in the DC of the intestinal lamina propria corresponds to an increase of IL-17-producing CD4+ T-cells [116].

Notch 2 double deficient animals disclose differentiation of naïve CD4+ T-cells towards Th2 cells [120]. In Th1 differentiation, the role of Notch 2 signaling is less clear, as some studies showed that Th1 differentiation is increased by the Notch family members without specific details and focuses attention on the role of the two ligands Delta1 and Delta4 [121]. However, Th1 cell response is also present in Notch 2, Notch 1 and RBP-J knocked-down mice [120]. This observation leads to the hypothesis that the development of Th1 is regulated by several members of the Notch family or by all of them in a redundant way.

Maekawa Y. and co-workers [122] showed that Notch 2 signaling is able to promote cytotoxic T-cells (CTLs). In fact, in mice, Notch 2-deficient T-cells altered differentiation into CTLs and DCs with low expression of the ligand DLL1 that caused a less efficient differentiation of CTLs [122]. The intracellular domain of Notch 2 interacts with the cAMP responsive element binding protein 1 (CREB-1) [123] that binds a DNA nucleotide sequence present in many cellular promoters stimulating transcription. The control of T-cell cytotoxicity depends on the combining of Notch 2 and CREB1 signals [122].

The CTLs are involved in cancer immunosurveillance, and Notch 2, unlike Notch 1, seems to be required for the efficient induction of their antitumoral activity [124].

It is well-known that Notch signaling is involved in the self-renewal of stem cells and in the growth and differentiation of progenitors in various organs. In mice, Notch 2 improved the formation rate of short-term repopulating multipotential progenitor cells (MPPs) as well as long term repopulating HSCs [125]. Varnum-Finney B. et al. demonstrated in their studies that a Notch 2-dependent role in ensuring repopulation by HSCs, MPPs and lymphoid cells is present during BM regeneration [125]. In fact, Witt et al. previously described a double role of Notch 2 in T and B lineage stem cells, artificially inducing various levels of an activated form of Notch 2 (N2IC) in murine hematopoietic progenitors [126]. While high levels of N2IC led to T-cell leukemia, lower levels boosted the maturation of the CD8 lineage. In contrast, concerning the B lineage, N2IC drove towards the development of the B1 B-cell subset [126]. The different expression levels of N2IC suggests that Notch 2 signaling modulation may have significant consequences for thymocyte development. In mice expressing high levels of N2IC, peripheral T-cell populations have high proliferation rates. Conversely, in mice with low levels of N2IC, no disease is diagnosed until month 10 after transplantation [126]. In addition, the endogenous Notch 2 protein is more expressed in the pro-B-cells, as their development requires a crosstalk with bone marrow stroma. As Notch 2 is expressed in both MZ B and B1 B-cells, but not in FO B or B2 cells, members of the NF-κB pathway, targeted by Notch signaling, may be involved in the development of both MZ B and B1 B-cells subsets. In summary, the effect of N2IC on the B-cell lineage confirms the involvement of Notch 2 signaling in the development of B-cell subsets [126].

The biologic effects of Notch 2 in the physiology of the immune system cells are described in Figure 2.

Considering the crucial role of Notch 2 in different aspects of the physiological development/maturation processes and homeostasis of immune system cells, it is not hard to understand how different alterations in its signaling could be involved in hematologic malignancies. These alterations are mostly somatic mutations determining gain-of-function with a potentiated Notch 2 transcriptional activity.

### 3.2. Notch 2 Mutations in B-Cell Lymphomas

The hyperactivation of Notch 2 through gain-of-function mutations in subtypes of mature B-cell neoplasms has been proven by Lee et al. [98]. These mutations were the partial or complete deletion of the PEST domain, or a single amino acid substitution at the C-terminus of the Notch 2 protein [98]. Notch 2 mutations were identified in DLBCL [98] and in MZB-cell lymphoma [99,100]. The PEST domain mutations cause an increased stability of Notch 2, prolonging its life. In fact, PEST sequences trigger the ubiquitin-mediated proteolysis [127] and mutations in this region, producing Notch 2 truncated forms, could prevent receptor degradation and increase its activity. The presence of recurrent somatic gain-of-function mutations in Notch 2 was also proven, using whole-genome DNA sequencing (WGS), by Kiel and colleagues [100]. They found two main types of mutations: alterations in the PEST domain and, more rarely, nonsynonymous substitutions affecting the extracellular HD [100]. Overall, only MZL (25% splenic and 5% non-splenic) showed Notch 2 mutations that were not detectable in other hematologic malignancies [100].

In addition, the authors compared mutated to wild type Notch 2 lymphomas regarding the clinical outcomes, reporting a shorter relapse-free survival, for cases carrying Notch 2 mutations [100]. Notch 2 mutations in Splenic MZL (SMZL) were identified by Rossi and colleagues as truncating events (14 frameshift indels and 11 nonsense mutations) and clustered within a hotspot region in exon 34, including a recurrent p.R2400 nonsense mutation in 24% of cases [92]. In the same study, Notch 2 mutations were described in a small percentage (3.7%) of DLBCL [92]. These observations led to a further investigation focused on this high-grade histotype [93]. The authors evaluated HCV-positive and negative DLBCL and observed a frequency of 25% in mutations in the Notch 1 and Notch 2 genes in the HCV-positive samples while only in 1% of the DLBCL negative for HCV [93]. Since these mutations were associated with a severe prognosis and with the presence of low-grade components in the diagnostic biopsy, the authors suggested that at least a fraction of the HCV-positive DLBCL could represent the transformed phase of an MZL clone or the coexistence of high and low-grade components [93]. A similar pathogenetic hypothesis was previously and cautiously suggested by Lee et al. who did not find Notch 2 mutations in MZB-cell lymphomas, but proposed the interesting possibility that some or all the DLBCL cases carrying Notch 2 mutations were transformed from MZB-cell lymphoma [98].

As already mentioned, PEST domain somatic mutations were also described for high-grade forms such as the DLBCL [98]. In this setting, a genome-wide approach revealed a rarer copy number increase of the mutated Notch 2 allele [98], a phenomenon previously associated with oncogenic gene alterations in other cancers [128].

Overall, Notch 2 mutations are involved in the pathogenic processes of MZL and DLBCL. Although the fine mechanisms still need to be understood, these alterations could represent a hypothetical biomarker and in addition the Notch 2 mutations could help inform SMZL diagnosis and prognosis [129].

### 3.3. Notch 2 in B-Cell Acute and Chronic Lymphocytic Leukemia

Acute myeloid leukemia (AML) is the most common myeloid malignancy in adulthood, characterized by the impairment of myeloid differentiation and blast-cell accumulation in the BM [130]. Chemo-resistance is a not rare feature of AML cells, usually due to the persistence of residual blast-cells in the BM. Signals coming from stromal cells could be responsible for inducing these kinds of abilities in AML cells through the involvement of different pathways, including Wnt/β-catenin and Notch [110].

In detail, in AML the Notch 2 pathway is boosted by receptor overexpression. Such an overexpression is not caused by a mutational event but by an altered microenvironmental signaling from bone-marrow mesenchymal stromal cells [101] inducing AML cell survival and chemo-resistance [101].

In their study on CLL, Mangolini et al. [102] used an opposite approach, based on the same concept that malignant B-cells do not survive nor proliferate autonomously, but rather in conjunction with tumorigenic cues from the stromal microenvironment. Since CLL cells constitutively express the Notch ligands JAG1, JAG2 and DLL, the authors evaluated the expression and activation of Notch 1 and Notch 2 in stromal cells [102]. They found a double role for stromal Notch 2 activation. Notch 2 activation regulates N-cadherin expression in CLL cells, interacting with and further stabilizing β-catenin and, at the same time, it boosts canonical Wnt signaling in the same CLL cells [102]. All these effects were reverted by the pharmacological inhibition of the Wnt pathway as well as the inhibition of Notch 2 in stromal cells [102].

Sera from patients with chronic B-CLL contain high amounts of soluble CD23 (sCD23), which reflects disease activity and is an important indicator of disease progression as confirmed by numerous reports [103].

CD23, which usually is a transitionally expressed marker of B-cells, is a downstream target of Notch 2 signaling [104,105]. The membrane CD23 undergoes spontaneous proteolysis, producing abnormal levels of soluble (sCD23) molecules in CLL. In this study, Hubmann et al. demonstrated a direct link between Notch 2 signaling ‘‘gain-of-function’’ and the higher levels of CD23 and in a further study, showed that peripheral CLL cells overexpressed a transcriptionally active form of Notch 2 [105]. This transcriptionally active Notch 2 truncated form acts independently from the proteolytic cleavage of γ-secretase complex that cuts the intracellular domain of the wild type Notch making it able to translocate to the nucleus. Thus, GSIs are putative novel anticancer agents at least in some Notch -associated malignancies [105]. These limitations could be overcome by a molecule selectively inhibiting all the Notch 2 isoforms, namely gliotoxin that promotes the induction of apoptosis in CLL cells contrasting the negative effects of stromal crosstalk [131,132] (Figure 1).

## 4. Notch 3

### Notch 3 from Physiological to Oncogenic Pathways

Notch 3, together with Notch 4, is the least studied of the four Notch receptors, although it has been implicated in the biology of several solid cancers including melanoma, lung, breast, hepatocellular-, prostatic-, ovarian- and cholangiocarcinomas [133,134,135].

As the roles of Notch 3 and Notch 4 are not yet fully understood, we will focus on the physiology and the signaling alterations leading to hematological malignancies in the same paragraph.

The Notch 3 biologic effects in the physiology of the immune system cells are summarized in Figure 2.

Notch 3-driven oncogenic pathways involve the main aspects of tumorigenesis such as the inhibition of apoptosis, neoangiogenesis and metastatization.

Inhibition of apoptosis plays a critical role in tumor cell survival and development. In this respect, Notch 3 is able to modulate programmed cell death by activating the Bcl-2 protein family [136,137]. The ability of Notch 3 to promote cancer cell survival has also been seen in hematological malignancies such as B-Acute Lymphoblastic Leukemia (B-ALL) [106,138,139].

In detail, once again, the crosstalk with stromal cells is able to sustain the abnormal viability of B-cells in B-ALL: a stromal cell-mediated antiapoptotic effect on B-ALL cells mediated by Notch 3 and Notch 4 or JAG1/2 and DLL1 in a synergistic manner was described [106].

Notch 3 together with Notch 1, is involved in T lymphocyte development [140]. Its expression starts during embryogenesis and increases over time during fetal development in the thymus cortex, where the T-cells mature [140]. Notch 3 overexpression induces pre-T-cell receptor expression in immature T lymphocyte [141]. This was also confirmed by a flow cytometry analysis in mice that found a high Notch 3 expression in immature thymocytes that decreased during positive selection [142]. Furthermore, a study by Suliman and collaborators confirmed that Notch 3, together with Notch 1, participates to the physiological thymocyte β-selection [143]. Notch 3 sustains survival, proliferation and the invasion of lymphoma cells via different pathways [144,145]. Notch 3-activating mutations in the non-regulatory and PEST region are correlated with a high level of the intracellular domain in T-ALL human cell line lacking Notch 1 mutations. This confirms the role of Notch 3 signaling in T-ALL [107] and the fact that Notch 1 and Notch 3 effects are redundant. In fact, Notch 1 and Notch 3 share similar oncogenic pathways in T-ALL, inducing the expression of the same genes since they bind the same DNA sites [146]. Tottone et al. described a histonic demethylation/methylation mechanism responsible for regulating Notch 3 and Notch 1 expression in T-ALL [147]. Pharmacological inhibition of the two involved enzymes decreased the expression levels of Notch 3, Notch 1 and Notch targets, abrogating T-ALL cell viability. Therefore, it is conceivable that a specific enzymatic inhibition has potential therapeutic anti-leukemic efficacy in Notch /c-Myc axis-related malignancies [147].

The strategy of targeting a histone deacetylase to turn off Notch 3 signaling is suggested by the results of Pinazza et al., as Notch 3 lysosome-dependent degradation is increased when a deacetylase called HDAC6 is inhibited in vitro and in vivo [148].

Notch 3 mutations were also described in blastoid and pleomorphic mantle cell lymphomas, an aggressive variant of mantle cell lymphoma [89]; although the study was meant as a genomic analysis and provided a genomic profile without functional hypotheses, the Notch 3 mutation was found in aggressive forms with high levels of Ki-67 protein levels, a protein strictly associated with cell proliferation [89]. In line with these findings, a whole genome sequencing of ovarian DLBCL tissue with a high Ki-67 index revealed the presence of missense mutations both in Notch 3 and Notch 4 genes [149].

As previously mentioned, CXCR4-receptor facilitates cell migration in Notch 1-induced T-ALL models [65]. A study by Ferrandino et al. showed that Notch 3 positively regulated CXCR4 on the surface of human leukemia cells promoting the anomalous migration of immature T-cells from the thymus in early leukemia stages [108].

Another key point in tumorigenesis is that, once an angiogenic switch is turned on, cancer begins to grow and metastasize, and therefore the discovery of new antiangiogenetic drugs is one of the most promising targets in oncology. The role of angiogenesis in the growth and survival of hematological malignancies has been evident since 1994 [150] in a series of experiments showing that tumor progression is clearly related to its degree of angiogenesis [151].

The Notch pathway contributes to endothelial cell-to-cell communication and is essential for functional vessel patterning and remodeling during development and adulthood [9]; more recently, an emerging picture is that Notch receptors coordinate vascular endothelial growth factor (VEGF)–mediated angiogenesis [9]. Notch 3 acts as a critical regulator of pathological blood vessel formation and its abnormal activation spurs neoangiogenesis in different cancers as stated by several research groups but no data are available concerning NHLs [139]. The ability of a cancer cell to spread, promoting metastases, is considered as a key step in cancer progression and is associated with a poor prognosis and poor overall survival. Research has shown that Notch 3 is associated with a higher invasiveness and metastatic rate in many cancer types [139]. Furthermore, high Notch 3 levels correlated with a poor clinical outcome, a diminished disease-free and overall survival [139,152] and chemo-resistance [110,139].

In addition, Notch 3 was identified in DLBCL samples as alternatively spliced, with differential exon 16 depletion (-exon 16) among different subtypes [109]. In fact, predicted vincristine resistant patients from the germinal center subclass had significantly downregulated Notch 3 -exon 16 transcript expression and generally displayed adverse overall survival in R-CHOP treated patients [109]. Therefore, specific alternatively spliced Notch 3 differentiated molecular subtypes of DLBCL, with a prognostic and predictive biomarker potential in germinal center subtypes was found [109].

We also consulted multiple atlas to gather information regarding Notch 3 expression profiles in lymphomas. The Cancer Genome Atlas (TCGA) (https://www.cancer.gov/about-nci/organization/ccg/research/structural-genomics/tcga), a landmark cancer genomics program, reported that Notch 3 mRNA is 14–15 times more expressed in Non-Hodgkin Lymphomas (NHL) lymphocytes compared to normal B-cells as well as the “Human Protein Atlas” which confirmed a medium-high expression of Notch 3 receptor in lymphoma tissue versus a low expression in healthy controls (https://www.proteinatlas.org/).

In summary, compelling evidence points to Notch 3 as a pleiotropic regulator of hematological cancers biology although this promising field of research is still in the early stages and merits further investigation.

## 5. Notch 4

### Notch 4 from Physiological to Oncogenic Pathways

Notch 4 is a divergent member of the Notch receptors and is mainly expressed in the vasculature [153], in human bone marrow cells and in CD34^+^ and CD34^−^ populations [14,154] (Figure 2).

Some somatic mutations of Notch 4 have been described. In fact, a truncated variant of Notch 4 named Int-3 is constitutively active and was first identified as an endothelial cell-specific mammalian Notch gene [155]. An in vitro study with Int-3-transduced human myeloid leukemia HL-60 cell line showed that Notch 4 signaling inhibits differentiation and promotes the expansion of hematopoietic stem/progenitor cells [112].

Notch 4 intracellular domain-transduced cord cells transplanted into mice showed remarkably elevated levels of engraftment derived from an immature T-cell population, while B-cell development was inhibited [156]. Taken together, these results indicate that the activation of Notch 4 (or constitutively active Notch 4 variants) leads to enhanced stem cell activity, reduced differentiation and altered T and B lymphoid development [156].

Furthermore, a few indicators suggest that the bone marrow stromal environment could mediate Notch 4 signaling (together with Notch 1 and 2, as previously mentioned) in CLL, determining survival and resistance to chemotherapy [110]. The same authors highlighted the role of stromal cells in inducing an anti-apoptotic effect on B-ALL cells, mediated by Notch 3 and 4 or JAG1/2 and DLL1 in a synergistic manner [106].

Furthermore, a Notch 4 germline single nucleotide polymorphism (SNP) was reported as associated to an HCV-related lymphoproliferative disorder called cryoglobulinemic vasculitis [157]. The minor allele of the Notch 4 SNP determined an increased risk of vasculitis in HCV chronically infected patients [157]. On this basis, another study confirmed the significant relationship between this allelic variant and an increased lymphoma risk in a group of patients with HCV-related lymphomas, suggesting Notch 4 polymorphism as a possible non-invasive marker of hematological malignancy [111]. An association of a Notch 4 intronic SNP and the risk of disease relapse in patients with leukemia after transplantation was found in a genomic analysis on 141 patients with leukemia and their respective hematopoietic stem cell donors [158]. This latter association analysis suggests unknown pathophysiological functions of Notch 4 SNPs in hematological malignancies, thus requiring future investigation to ascertain their biological effects.

## 6. Conclusions

An increased Notch-induced signaling is involved in different hematologic malignancies.

Several authors stressed the role of bidirectional interactions between tumoral cells and their environment, sustaining cancer progression and inducing aggressive features. This seems to be particularly relevant for bone marrow stromal cells that largely contribute to the survival of leukemia cells via Notch/Notch ligand signaling. As seen in other kinds of cancers, regulating the crosstalk between the different compartments of the bone marrow elements could represent an innovative therapeutic perspective. In fact, experimental drugs capable of interrupting Notch signaling were tested in phase I clinical trials. Among them, the most promising were the γ-secretase inhibitors, although, so far, they have shown a clinical efficacy lower than expected and several side effects. Another possible approach is represented by the use of anti-Notch monoclonal antibodies blocking the external binding site. This could be theoretically effective when an increased signaling is sustained by the tumoral microenvironment (i.e., through ligand overexpression) or in the case of a Notch-mutated extracellular domain.

The increased Notch signaling activation could be due to somatic mutations producing constitutively activated forms of the receptors. As this happens, the malignant-cells are free from microenvironment control and, therefore, proliferation and other tumoral features are self-promoted. In this case, the implementation of a potential therapeutic strategy seems to be more complex, even if a mutational pattern could be used as genetic profiling to identify more aggressive forms and to select a more tailored method of treatment.

Overall, Notch pathway alterations seem to have pleiotropic activities in hematological malignancies, contributing to different aspects of tumorigenesis and cancer progression. While Notch 1 contribution is a major concern for T-cell diseases, Notch 2 plays a more important role in B-cell disorders, taking into account its differential involvement in T and B-cell physiology.

More studies are needed to clarify how Notch 3 participates in hematological malignancy pathogenesis, while the lack of compelling data on Notch 4 possibly means a less direct or a non-canonical involvement in leukemia and lymphoma. Further analyses are vital in order to ascertain the role of the last two Notch family members and to more accurately define the effects of the first two in hematological malignancies and to implement safe and effective new therapies.

## Figures and Tables

**Table 1:** Involvement of all Notch receptors in B and T-cells malignancies.

Notch Receptor	Tumor Type	Type of Alterations	Ref
**NOTCH 1**	RS	30% of cases carry Notch 1 genetic alteration with overexpression and MYC activation	[13]
	T-ALL	T(7;9) is responsible for abnormal Notch 1 signaling	[42]
		Activating mutations in HD and PEST domain	[43]
		Presenilin-1, a γ-secretase, is overexpressed in T-ALL cells	[49]
		FBXW7 mutations avoid NICD degradation sustaining Notch 1 signaling	[57]
		NICD binds to NF-kB promoters increasing protein expression	[71]
	B-CLL	Constitutively expressed, confers resistance to apoptosis	[75]
	CLL	Somatic mutations generate stable and activated forms of the protein and are associated with advanced tumor stage	[76]
		Mutation in FBXW7 reduce degradation of Notch intracellular domain	[81]
	MM	Notch 1-JAG1 interaction resulted in apoptosis protection	[82]
		DLL4 interaction in CLL with activating Notch 1 mutations promotes proliferation, migration and angiogenesis	[83]
	MCL	Notch 1 mutations associated with severe prognosis	[86,87,89,90]
		Notch 1 pathway overexpression by DLL4 interaction in patients with Notch 1 mutation	[88]
	ALCL	Mutation in extracellular domain enhance cell proliferation	[91]
	SMZL	Notch 1 mutations were present in 5% of cases	[92]
	DLBCL	Genetic Notch 1 alteration in small subset of HCV positive patients are associated with a worse prognosis	[93]
	MALT	Notch 1 mutations were found in some cases but further analyses are needed to explain biological meaning	[95,96]
	FL	Mutations are associated with splenic involvement	[97]
**NOTCH 2**	DLBCL	Mutated PEST domain is associated with severe prognosis	[93,98]
	MALT	Notch 2 mutations were found in some cases but further analysis is needed to explain biological meaning	[95]
	FL	Mutations are associated with splenic involvement	[97]
	MZB	Activating mutations are associated with poor outcomes	[99,100]
	AML	Overexpression promotes cell survival and chemo-resistance	[101]
	CLL	Notch 2 boosts Wnt signaling in stromal cells	[102]
	B-CLL	High concentration of soluble CD23 by Notch 2 signaling promotes disease progression	[103,104,105]
**NOTCH 3**	MCL	Mutations are associated with severe prognosis in variants with high levels of Ki-67, a protein associated with proliferation	[89]
	T-ALL	Increased crosstalk with stromal cells mediating an antiapoptotic effect on leukemia cells	[106]
		Activating mutation in non-regulatory region and PEST domain enhanced Notch 3 signaling	[107]
		Notch 3 boosts CXCR4 expression and promotes cell migration	[108]
	DLBCL	An alternatively spliced variant, with differential exon 16 depletion (-exon 16) was described among different subtypes	[109]
**NOTCH 4**	CLL	Stromal cell stimulation of Notch 4 determines survival and resistance to chemotherapy	[110]
	B-ALL	Stromal cell induces an anti-apoptotic effect	[106]
	NHL	Notch 4 germline single nucleotide polymorphism was reported as associated to an HCV-related NHL	[111]
	DLBCL	A truncated variant of Notch 4 named Int-3 is constitutively active in human myeloid leukemia HL-60 cell line inhibiting differentiation and promoting the expansion of hematopoietic stem/progenitor cells	[112]

## Abbreviations

T-ALL	acute lymphoblastic leukemia
B-CLL	B-cell chronic lymphocytic leukemia
CLL	B-cell chronic lymphocytic leukemia
DLL4	Delta-like ligand 4
MCL	mantle cells lymphoma
RS	Richter Syndrome
ALCL	anaplastic large cell lymphoma
MM	multiple myeloma
SMZL	splenic marginal zone lymphoma
DLBCL	diffuse large B-cell lymphoma
MALT	mucosa-associated lymphoid tissue
FL	follicular lymphoma
MZB	marginal zone B
AML	acute myeloid leukemia
NHL	Non-Hodgkin Lymphoma
